# Two-stage revision for periprosthetic joint infection in cemented total hip arthroplasty: an increased risk for failure?

**DOI:** 10.1007/s00402-022-04671-3

**Published:** 2022-11-03

**Authors:** Christian Hipfl, Vincent Leopold, Luis Becker, Matthias Pumberger, Carsten Perka, Sebastian Hardt

**Affiliations:** Centre for Musculoskeletal Surgery, Department of Orthopaedics, Charité Universitaetsmedizin Berlin, Charitéplatz 1, 10117 Berlin, Germany

**Keywords:** Periprosthetic infection, Revision total hip arthroplasty, Two-stage revision, Fixation, Cemented, Cementless, Reinfection

## Abstract

**Background:**

The impact of the prior fixation mode on the treatment outcome of chronic periprosthetic joint infection (PJI) of the hip is unclear. Removal of cemented total hip arthroplasty (THA) is particularly challenging and residual cement might be associated with reinfection. This study seeks to compare the results of two-stage revision for PJI in cemented and cementless THA.

**Methods:**

We reviewed 143 consecutive patients undergoing two-stage revision THA for PJI between 2013 and 2018. Thirty-six patients with a fully cemented (*n = *6), hybrid femur (*n = *26) or hybrid acetabulum (*n = *4) THA (cemented group) were matched 1:2 with a cohort of 72 patients who underwent removal of a cementless THA (cementless group). Groups were matched by sex, age, number of prior surgeries and history of infection treatment. Outcomes included microbiological results, interim re-debridement, reinfection, all-cause revision, and modified Harris hip scores (mHHS). Minimum follow-up was 2 years.

**Results:**

Compared with PJI in cementless THA, patients undergoing removal of cemented THA had increasingly severe femoral bone loss (*p = *0.004). Patients in the cemented group had an increased risk for positive cultures during second-stage reimplantation (22% compared to 8%, *p = *0.043), higher rates of reinfection (22% compared to 7%, *p = *0.021) and all-cause revision (31% compared to 14%, *p = *0.039) compared to patients undergoing two-stage revision of cementless THA. Periprosthetic femoral fractures were more frequent in the group of patients with prior cementation (*p = *.004). Mean mHHS had been 37.5 in the cemented group and 39.1 in the cementless group, and these scores improved significantly in both groups (*p < *0.01).

**Conclusion:**

This study shows that chronic infection in cemented THA might be associated with increased bone loss, higher rates of reinfection and all-cause revision following two-stage revision. This should be useful to clinicians counselling patients with hip PJI and can guide treatment and estimated outcomes.

## Introduction

The outcome of two-stage revision total hip arthroplasty (THA) for periprosthetic infection (PJI) is still unpredictable in some cases and literature shows reinfection rates of up to 30% [1–15]. It is well-studied that successful treatment depends on the causing pathogen, host and local tissue factors and chronicity of infection [1, 9, 14, 16–18]. However, the actual reconstruction techniques are often overlooked and their significance on the overall management of PJI has not been adequately investigated.

Traditionally, THA reimplantations have been carried out using cemented components with antibiotic-loaded bone cement [19–21]. However, long-term results of revision THA with cemented fixation have been associated with high rates of loosening [19, 20, 22]. The potential of biological fixation and improved implant designs have led to an increasing worldwide use of cementless components in revision THA including two-stage exchange procedures for PJI. Studies have shown promising long-term durability without compromising infection eradication rates [2, 8, 23–25]. Besides potential of long-term stability, it is also important to consider possible failure and further need for revision. Different fixation techniques may lead to changes in revision patterns as well as have effects on bone loss, which might influence the risk for reinfection, and vice versa [26–28]. Complete removal of a well-fixed femoral cement mantle can be challenging, and residual cement may act as a source of infection persistence [1, 29]. Aggressive debridement of the femoral cavity may result in loss of bone stock and femoral perforation or fracture [26, 28]. To our knowledge, the degree to which any of this might be the case in two-stage revision THA for infection has not been evaluated.

In this retrospective case–control study, we wanted to investigate the role of the previous fixation mode on survival following two-stage exchange THA by comparing the risk of reinfection and aseptic failure between patients who had infection of a cemented and cementless THA.

## Patients and methods

### Study design

After obtaining approval from our institutional review board, we reviewed our institutional database for the period January 2013 to March 2018 to identify 143 consecutive patients who underwent an entire two-stage exchange arthroplasty for hip PJI. Patients were stratified according to the fixation mode, which was present at the time of revision. We identified a study cohort of patients with a fully cemented (*n = *6), hybrid femur (*n = *26) or hybrid acetabulum (*n = *4) THA (cemented group: 36 hips / 36 patients). The control group consisted of patients who underwent two-stage exchange of a cementless THA and was matched for sex, age, number of previous surgeries and history of infection treatment at a 1:2 ratio (cementless group: 72 hips / 72 patients). Sex and previous history of infection treatment were matched exactly, whereas patient age and number of prior surgeries were matched as closely as possible. Patients with megaprostheses and a follow-up less than 24 months were excluded. All patients had surgical and antimicrobial treatment according to a standardized algorithm by a multidisciplinary team of orthopedic surgeons, infectious diseases physicians and microbiologists [30–32]. Medical records were reviewed for all details on demographics, comorbidities, host and extremity grades as described by McPherson et al. [16], American Society of Anesthesiologists (ASA) scores, operative characteristics and postoperative follow-up.

### Diagnosis and treatment

Diagnosis of PJI was based on the definition reported by Zimmerli et al. [30, 33], which included the confirmation of at least one of the following criteria: purulence around the prosthesis or a sinus tract; increased synovial fluid leucocyte count or differential (> 2000/µl leucocytes or > 70% granulocytes); confirmatory microbial growth in synovial fluid, periprosthetic tissue (≥ 1 specimen in highly virulent organisms or ≥ 2 specimens in low virulent pathogens) or sonication culture of retrieved components (> 50 colony-forming units (CFU)/mL sonication fluid [34]; or positive histopathology, defined as a mean of ≥ 23 granulocytes per 10 high-powered fields [35]).

All operations were performed by five senior surgeons specialized in total joint arthroplasty with experience in revision THA. During the first-stage procedure, a meticulous removal of all components, cement, plug and all other foreign material was performed. The removal of the femoral cement mantle was performed using chisels, curettes, and drills under fluoroscopic control. A cortical bone window and extend trochanteric osteotomy (ETO) was utilized in six patients (17%) and four patients (11%), respectively. Five periprosthetic tissue samples were collected and synovial fluid was aspirated for microbiological analysis. The components were sent for sonication. Thereafter, a thorough irrigation and debridement of bone and soft tissue was performed using a polyhexanide-containing solution. No cement spacer was implanted, and the wound was closed routinely in layers over a passive drain. Second-stage reimplantation was performed when the local status was satisfactory (surgical wound healed, no drainage, redness or increased swelling), laboratory signs of infection control (continuously decreasing C-reactive protein) were present, and the general status of the patient was suitable. Any evidence of persistent infection led to interim re-debridement. The decision to perform a re-debridement was made on the basis of clinical features, laboratory parameters and intraoperative findings and was surgeon dependent. During reimplantation, a renewed debridement including sample collection was performed.

After first-stage surgery, intravenous antibiotics were administered for 2 weeks followed by oral antibiotics until reimplantation. Between stages, patients received ongoing antimicrobial treatment. No drug holidays or diagnostic hip aspiration was done prior to reimplantation. After second-stage reimplantation, intravenous antibiotics were administered for 2 weeks followed by oral antibiotics for a minimum of 4 weeks. In case of confirmatory microbiological results at reimplantation (≥ 2 positive specimens, polymicrobial growth, or ≥ 1 positive specimen, if the isolated microorganism was the same as the initial infecting pathogen or a new highly virulent organism), antimicrobial treatment was extended from 6 to 12 weeks postoperatively.

### Radiographic analysis

Radiographic analysis was performed by a trained consultant-level orthopedic surgeon specializing in hip arthroplasty and an orthopedic surgery resident for all anteroposterior and lateral hip radiographs. Acetabular and femoral bone loss was classified according to the systems outlined by Paprosky et al. [36] and Della Valle and Paprosky [37]. Signs of implant loosening were determined using the system outlined by Harris and McGann [38]. All complications or other observations were recorded. For all radiographic analyses, a standardized measurement technique was ensured through teaching sessions in which interpretation of radiographic features was discussed between the observers.

### Outcome measures

Primary outcome of interest included revisions for reinfection and aseptic failure, all-cause revision, and complications. Infectious diseases physicians were consulted to help identify reinfections. Reinfection was defined as having at least one positive criterion according to the Zimmerli diagnostic criteria obtained through a joint aspirate or revision surgery during the follow-up period. Clinical outcomes including the modified Harris Hip Score (mHHS) [39] were also analyzed.

### Statistical analysis

Descriptive statistics are reported as number (percentage) or mean (range), as appropriate. Continuous variables were compared using the Mann–Whitney *U* test and categorical variables using the Chi-square test. Kaplan–Meier survivorship was calculated by using revision for reinfection, aseptic failure and all-cause revision as an end point. Survival comparisons between the cemented and cementless group were made with use of the log-rank test. Calculations were performed using SPSS version 25 software (SPSS Inc., Chicago, IL, USA). A *p* value < 0.05 was considered statistically significant.

## Results

### Demographics

The cemented group consisted of 20 females and 16 males with a mean age of 71.3 years (32.2–83.3) and the mean body mass index (BMI) was 29 kg/m^2^ (20–46). Besides the matched parameters, there were no significant differences in baseline demographics including McPherson host and extremity grade between the cemented and cementless group (Table 1). The mean prosthesis-free interval was 9.2 weeks (2.7–23.0) and 8.9 weeks (2.0–29.0) in the cemented and cementless group, respectively (*p = *0.817). Patients undergoing removal of cemented THA had increasingly severe femoral bone defects (*p = *0.004) and more frequently presented with radiographic loosening of the femoral component (*p = *0.023). All operative characteristics are summarized in Table 2. Two patients died in the cemented group and four patients in the cementless group until the latest follow-up (*p = *1.000). The mean follow-up was 5.3 years (3.0–7.5) and 5.6 years (3.0–7.5) in the cemented and cementless group, respectively (*p = *0.427).

**Table 1:** Demographic data comparing patients who had two-stage revision for PJI in cemented and cementless THA

Variable	Cemented^a^ (*n = *36)	Cementless (*n = *72)	*p* value
Sex (M:F)	16:20	32:40	1.000
Age at first-stage (years)	71.3 ± 8.8	70.0 ± 7.9	0.464
BMI (kg/m^2^)	29.2 ± 5.6	28.6 ± 5.5	0.603
ASA score			0.326
1	0 (0%)	2 (3%)	
2	16 (44%)	39 (54%)	
3	20 (56%)	31 (43%)	
McPherson host grade			0.867
A	7 (19%)	17 (24%)	
B	21 (58%)	41 (57%)	
C	8 (22%)	14 (19%)	
McPherson extremity grade			0.302
II	27 (75%)	60 (83%)	
III	9 (25%)	12 (17%)	
Diabetes mellitus	11 (31%)	15 (21%)	0.265
Corticosteroid use	3 (8%)	7 (10%)	0.814
Rheumatoid arthritis	3 (8%)	3 (4%)	0.373
Microbiology at first-stage			
Polymicrobial	12 (33%)	25 (35%)	0.866
Difficult-to-treat^b^	5 (14%)	7 (10%)	0.516
Negative cultures	2 (6%)	7 (10%)	0.460
Positive cultures at second-stage	8 (22%)	6 (8%)	**0.043**
Weeks between stages	9.2 ± 4.0	8.9 ± 4.9	0.817
Follow-up (months)	64.1 ± 21.4	67.1 ± 16.2	0.427

Means and standard deviations are reported, and *p* values were calculated either from chi-square test or Mann–Whitney *U* test; bold—the significance level was *p < *0.05

*PJI* periprosthetic infection; *THA* total hip arthroplasty; *BMI* body mass index; *ASA* American Society of Anesthesiologists

^a^Cemented or hybrid total hip arthroplasty

^b^Pathogens, for which no biofilm-active antibiotics exist (rifampin-resistant staphylococci, enterococci, ciprofloxacin-resistant gram-negative bacteria and fungi)

**Table 2:** Operative characteristics comparing patients who had two-stage revision for PJI in cemented and cementless THA

Variable	Cemented^a^ (*n = *36)	Cementless (*n = *72)	*p* value
Prior open surgical procedures	1.8 ± 1.4	1.6 ± 1.5	0.713
Revision for infection	18 (50%)	36 (50%)	1.000
Sinus tract present	7 (19%)	15 (21%)	0.866
Time from index THA (years)	9.3 ± 8.1	6.9 ± 6.8	0.113
Radiographic implant loosening			
Acetabular component	13 (36%)	25 (35%)	0.887
Femoral component	18 (50%)	20 (28%)	**0.023**
Paprosky bone loss, acetabular			0.749
1	5 (14%)	10 (14%)	
2A	7 (19%)	20 (28%)	
2B	5 (14%)	7 (10%)	
2C	10 (28%)	23 (32%)	
3A	3 (8%)	6 (8%)	
3B	6 (17%)	6 8%)	
Paprosky bone loss, femoral			**0.004**
1	9 (25%)	27 (38%)	
2	10 (28%)	34 (47%)	
3A	8 (22%)	7 (10%)	
3B	6 (17%)	4 (6%)	
4	3 (8%)	0 (0%)	
Split osteotomy	0 (0%)	3 (4%)	0.214
ETO	4 (11%)	26 (36%)	**0.006**
Cortical window	6 (17%)	0 (0%)	**0.000**
Duration of first-stage surgery (minutes)	167.2 ± 48.0	153.7 ± 56.3	0.220
Reimplanted components at second-stage			
Acetabular			0.538
Modular, porous-coated	5 (14%)	9 (13%)	
Highly porous metal	21 (58%)	51 (71%)	
Antiprotrusio cage	6 (17%)	8 (11%)	
Cemented	4 (11%)	4 (6%)	
Femoral			**0.003**
Extensively porous-coated	19 (53%)	56 (78%)	
Modular, fluted tapered	13 (36%)	16 (22%)	
Cemented	4 (11%)	0 (0%)	
Large diameter heads (≥ 36 mm)	31 (86%)	57 (79%)	0.381
Dual-mobility cups	7 (19%)	8 (11%)	0.238
Duration of second-stage surgery (minutes)	150.8 ± 50.7	153.7 ± 56.3	0.774

Means and standard deviations are reported, and *p* values were calculated either from chi-square test or Mann–Whitney *U* test; bold—the significance level was *p < *0.05

*PJI* periprosthetic infection; *THA* total hip arthroplasty; *ETO* extended trochanteric osteotomy

^a^Cemented or hybrid total hip arthroplasty

### Microbiology and reinfection

All pathogens leading to PJI are summarized in Table 3. In both groups, the most common microorganism was coagulase-negative *Staphylococcus* followed by *Staphylococcus aureus.* Besides a higher rate of *Escherichia coli* in the cemented group (*p < *0.001) and a higher rate of *Cutibacterium spp*. in the cementless group (*p = *0.026), there were no significant differences in the microorganism frequency between the two groups. Patients in the cemented group had an increased risk for positive cultures during second-stage reimplantation (odds ratio [OR] = 3.1; 95% confidence interval [CI] = 1.0–9.9; *p = *0.043).

**Table 3:** Comparison of microorganism frequency between cemented and cementless group

Isolated microorganism^b^	Cemented^a^ (*n = *36)	Cementless (*n = *72)	*p* value
Gram-positive bacteria			
Coagulase-negative *Staphylococcus* (sensitive)	21	41	0.891
Coagulase-negative *Staphylococcus* (resistant)	1	2	1.000
Methicillin-sensitive *Staphylococcus aureus*	5	5	0.241
Methicillin-resistant *Staphylococcus aureus*	2	3	0.746
Cutibacterium spp.	1	13	**0.026**
*Staphylococcus lugdunensis*	1	3	0.719
Viridans group *Streptococcus*	1	4	0.517
*Enterococcus faecalis*	2	6	0.603
*Enterococcus faecium*	2		**0.044**
*Peptostreptococcus micros*	1	2	1.000
*Finegoldia magna*		3	0.214
*Corynebacterium* spp.		3	0.214
*Actinomyces* spp.		1	0.478
*Peptoniphilus* spp.		1	0.478
*Cellulomonas*		1	0.478
Gram-negative bacteria			
*Escherichia coli*	6	0	**0.000**
*Enterobacter cloacae*		1	0.478
Polymicrobial	12	25	0.866
Negative culture	2	7	0.460

*p* values were calculated from chi-square test; bold—the significance level was *p < *0.05

^a^Cemented or hybrid total hip arthroplasty

^b^Includes preoperative and intraoperative cultures during first-stage surgery

**Fig. 1:**
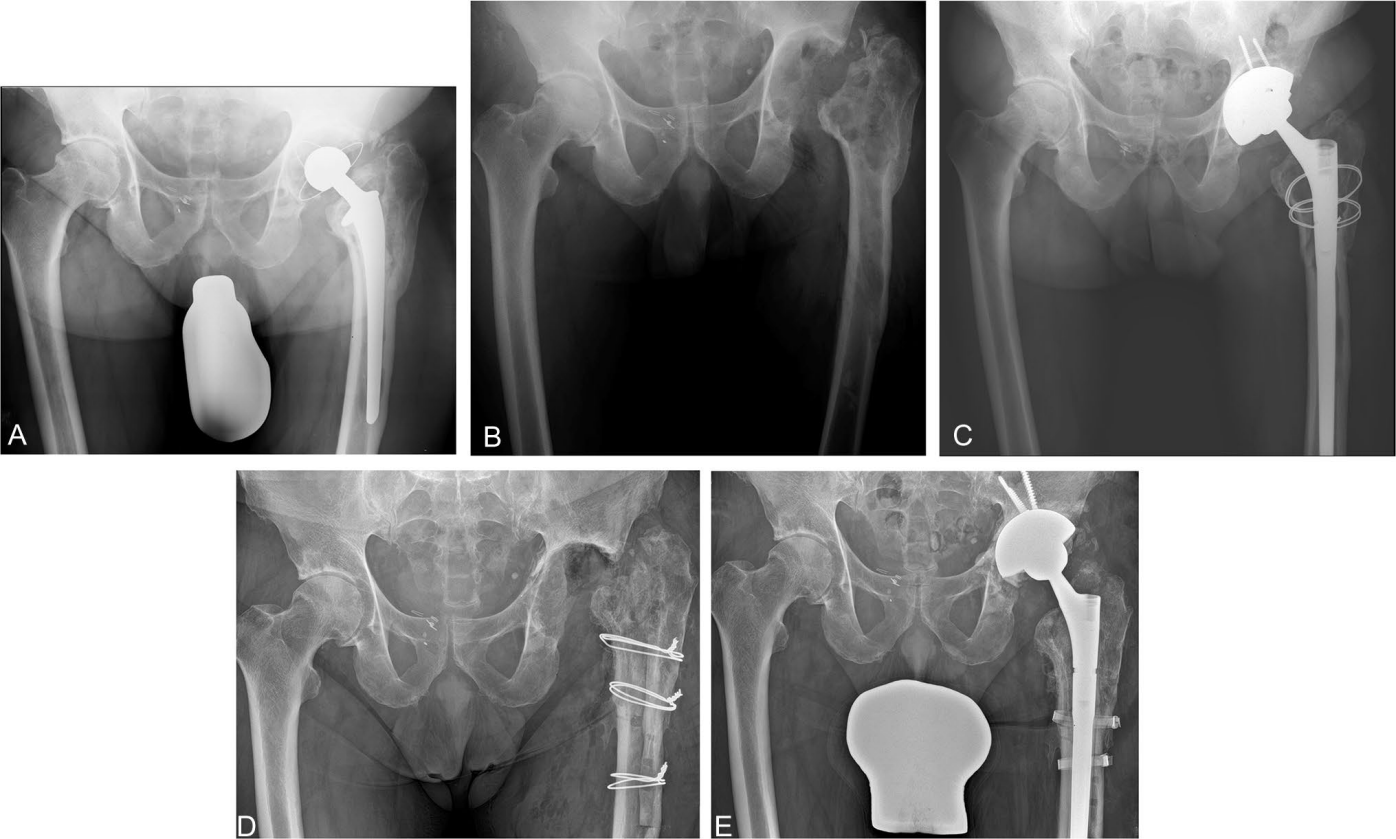
66-year-old male patient with chronic periprosthetic joint infection (PJI) of the left hip. **A** Anteroposterior view prior to two-stage exchange of the cemented total hip arthroplasty (THA) with loosening of the femoral component. **B** Radiograph following THA removal showing a Paprosky type 3B femoral defect and type 2B acetabular defect. **C** Radiograph showing the THA reimplantation after an interim period of 8 weeks using a highly porous metal shell and a modular, tapered fluted stem and two cerclage wires. **D** Radiograph following resection arthroplasty utilizing an extended trochanteric osteotomy after the patient suffered from reinfection at 35 months. **E** Radiograph showing the THA reimplantation again with a cementless revision stem; at 80-month follow-up the patient had no signs of PJI and a modified Harris hip score of 85 points

**Fig. 2:**
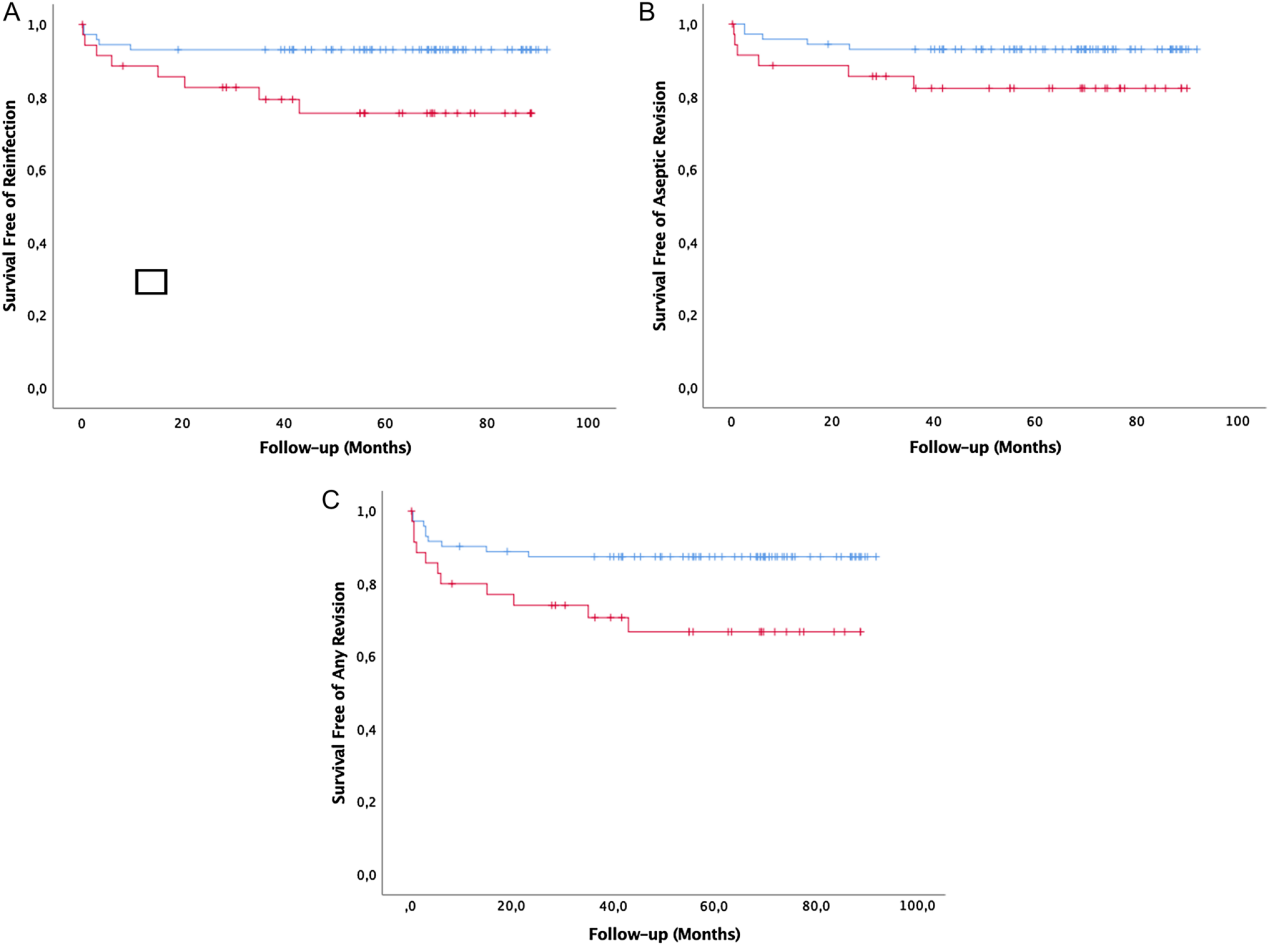
Kaplan–Meier survival curves for reinfection (**A**), revision for aseptic failure excluding reinfection treatment (**B**) and revision for any reason (**C**) following two-stage reimplantation. Patients with periprosthetic infection (PJI) in cemented or hybrid total hip arthroplasty (THA) (red) had overall five-year survival rates of 76%, 82% and 67%, respectively. Patients with PJI in cementless PJI (blue) had higher five-year survival rates of 93%, 93% and 87%

Cementation in earlier prosthesis was significantly associated with an increased risk of reinfection compared to two-stage revision of cementless THA (OR = 3.8; 95% CI = 1.2–12.7; *p = *0.021). Reinfection occurred in eight (22%) of the patients in the cemented group (Fig. 1) and five (7%) of the patients in the cementless group (Table 4). The mean time to diagnosis of reinfection following reimplantation was 15.1 months (0.3–43.0) in the cemented group compared with 3.8 months (0.5–9.7) in the cementless group (*p = *0.166). Kaplan–Meier survival estimates for reinfection in patients who were treated for infection of a cemented THA were 75.6% (95% confidence interval [CI]: 68.0%-83.2%) at 5 years with 16 hips at risk and compared with 93.1% (95% CI: 90.1%-96.1%) with 43 hips at risk in the cementless group (*p = *0.017) (Fig. 2a).

**Table 4:** Comparison of treatment outcome comparing patients who had two-stage revision for PJI in cemented and cementless THA

Variable	Cemented^a^ (*n = *36)	Cementless (*n = *72)	*p* value
Infection control			
Interim re-debridement	6 (17%)	7 (10%)	0.296
Reinfection after reimplantation	8 (22%)	5 (7%)	**0.021**
Other complications			
Femoral fracture in the interim period	1 (3%)	2 (3%)	1.000
Dislocation after reimplantation	6 (17%)	5 (7%)	0.128
Periprosthetic femoral fracture after reimplantation	4 (11%)	0 (0%)	**0.004**
Cup loosening	0 (0%)	1 (1%)	0.477
Stem loosening	1 (3%)	1 (1%)	0.613

*p* values were calculated from chi-square test; bold—the significance level was *p < *0.05

*PJI* periprosthetic infection; *THA* total hip arthroplasty

^a^Cemented or hybrid total hip arthroplasty

### Aseptic revision and other complications

Within the group of infected cemented THA, six patients (17%) required aseptic revision following reimplantation after a mean of 11.2 months (0.5–36.0) compared to five patients (7%) after a mean of 9.9 months (2.6–23.3) in the cementless group (OR = 2.7; 95% CI = 0.8–9.5; *p = *0.115). Patients who were treated for infection of a cemented THA had a higher rate of periprosthetic femoral fractures after reimplantation compared to patients with infection of a cementless THA (11% vs. 0%; *p = *0.004). One femoral component had to be revised for aseptic loosening in each group (*p = *0.613). The information of complications is summarized in Table 4. Kaplan–Meier survival estimates for all-cause revision in the cemented group were 66.8% (95% CI 58.5–75.1%) at 5 years with 14 hips at risk and compared with 87.4% (95% CI 83.5–91.3%) with 39 hips at risk in the cementless group (*p = *0.017) (Fig. 2c).

### Clinical outcome measures

The postoperative improvement in the mHHS score was significant in both groups at final follow-up (*p < *0.01). mHHS were available for 23 (64%) of 36 patients treated for PJI of a cemented THA at the latest follow-up and improved from 37.5 points preceding first-stage surgery to 64.1 points. The preoperative and postoperative mHHS did not differ between the groups (*p = *0.680 and *p = *0.795, respectively).

## Discussion

Cementless reimplantation is increasingly performed in two-stage exchange THA for infection due to good long-term survivorship and comparable eradication rates [2, 8, 23–25]. However, patients with chronic PJI often present with a history of infection treatment and different fixation methods. Changes in fixation technique may lead to changes in the outcome of the subsequent revision and more importantly might influence the risk of reinfection. To date, the influence of the prior fixation method on the outcome of two-stage revision THA is unclear. Considering that residual cement might be associated with infection persistence and revision of cemented THA might be related to greater bone loss, we hypothesized that two-stage revision of a cemented THA is associated with poorer outcome compared to the treatment of chronic PJI in cementless THA.

Our study showed that patients with chronic PJI of a cemented or hybrid THA were at elevated risk for reinfection, aseptic revision and all-cause revision following two-stage revision (Fig. 2). Patients requiring removal of cemented THA had more severe femoral bone defects compared to patients with infected cementless THA (*p = *0.004). The majority of patients in both groups had medium size femoral defects (Paprosky type 2 and type 3A), but large bone defects (Paprosky type 3B and type 4) were more frequently observed in the cemented group (cemented: 25% vs cementless: 4%; Table 2). This is in-line with the registry-based data for revision THA for any reasons, which also shows that patients with cemented stems have poorer bone stock at the time of revision [26, 28]. The patterns of bone loss associated with failed cemented THA may prejudice the results of future revision procedures [26]. To our knowledge, the underlying study is the first to document this in two-stage revision THA. In the past, most large cohort studies have not specified the type of fixation that was present at the time of infection. Some authors have found that retained cement is a risk factor for recurrent infection [1, 29, 40]. To our knowledge, the only contemporary cohort study of two-stage revision THA, which has analyzed the preexisting fixation mode could not found an association with increased reinfection rates [41].

Despite greatest efforts to perform a rigorous debridement, it can be difficult to guarantee complete removal of the femoral cement, even under fluoroscopy. Patients in the cemented group had a higher rate of interim re-debridement compared to patients with cementless THA (17% vs. 10%); however, this was not significant (*p = *0.296). Interestingly, patients with removal of cemented components more frequently showed positive cultures at the time of reimplantation (OR = 3.1; 95% CI = 1.0–9.9; *p = *0.043). These findings support the hypothesis that debridement in cemented THA is more difficult and unsuccessful than in cementless THA. Positive cultures at the time of reimplantation have been reported as a risk factor for reinfection [42, 43]. However, an association between prior cementation and an increased rate of positive cultures at the time of reimplantation has not yet been described.

Investigations on correlations between the microbial profile and fixation type are scarce. In our series, *Escherichia coli* was more frequent in the cemented group (*p < *0.001) and patients who had removal of cementless THA had a higher rate of *Cutibacterium spp.* (*p = *0.026). To our knowledge, higher rates of *Escherichia coli* infections in cemented prosthesis have not yet been reported. Recently, however, Hedlundh et al. also found an unexpectedly high number of *Cutibacterium acnes* infections in cementless primary THA [44]. The increasing role of *Cutibacterium acnes* as a true pathogen and not a commensal in PJI has previously been described [45]. The possible relationship with uncemented prosthesis is novel and should be confirmed by larger multicenter studies.

Apart from reinfection, patients with cemented or hybrid THA more frequently required revision for aseptic failures following two-stage revision THA compared to patients who had revision of infected cementless THA (17% vs. 7%), however this was not significant (*p = *0.089) (Fig. 2b). Postoperative femoral fractures were seen in four patients, of which all occurred in the cemented group (*p = *0.004). This higher risk of postoperative fractures may be explained by the fact that removal of the cement mantle, especially in endofemoral revision, may not be performed consistently resulting in unnoticeable weakening of the femoral cortex. On the other hand, patients with cemented THA more often presented with severe femoral bone defects, which could also explain the higher fracture rate.

Finally, clinical outcome scores measured with the mHHS improved significantly with no differences between the two groups. Overall, scores were comparable to other studies reporting on two-stage revision THA for infection [2, 8, 23, 24] and were lower than reported scores after revision THA for other etiologies (e.g., aseptic loosening) [46].

Our study had several limitations, and our findings should be interpreted in the light of these issues. First, this was a retrospective analysis, which has inherent drawbacks. Second, the sample size was relatively small, and thus the study was likely underpowered. However, treatment protocols have evolved over the last decade and this study depicts a consecutive series from a single institution using a stringent two-stage protocol with only minor variations. Lastly,  operative characteristics and microbial profiles had not been evenly distributed between groups. This may result in bias. Given the small number of cases, we were unable to examine the influence of all potential confounding factors. However, due to the limited information available in the literature, we believe our findings to be important. The comparative nature of the study including 2:1 matching including similar number of prior surgeries as well as McPherson host and extremity grades within the groups adds strength to the findings.

This study shows that patients who present with chronic PJI in cemented THA are more likely to have greater femoral bone loss. Two-stage revision of cemented implants was associated with higher rates of reinfection and revision for any reason. Aseptic failures were mostly due to periprosthetic fractures and dislocations. Long-stemmed, diaphyseal-engaging implants showed good durability at the mid-term, with comparable functional outcomes, independent of prior cementation. Large-scale randomized controlled trials are required to confirm our results.

## Data Availability

Data generated during and/or analysed during the current study are available from the corresponding author on reasonable request.
